# Implications of the syntheses on definition, theory, and methods conducted by the Response Shift – in Sync Working Group

**DOI:** 10.1007/s11136-023-03347-8

**Published:** 2023-02-09

**Authors:** Mirjam A. G. Sprangers, Richard Sawatzky, Antoine Vanier, Jan R. Böhnke, Tolulope Sajobi, Nancy E. Mayo, Lisa M. Lix, Mathilde G. E. Verdam, Frans J. Oort, Véronique Sébille, Response Shift – in Sync Working Group

**Affiliations:** 1 Department of Medical Psychology, Amsterdam UMC, Location University of Amsterdam, Meibergdreef 15, J3-211, 1105 AZ Amsterdam, The Netherlands; 2 Amsterdam Public Health, Mental Health, Amsterdam, The Netherlands; 3 School of Nursing, Trinity Western University, Langley, BC Canada; 4 Centre for Health Evaluation and Outcome Sciences, University of British Columbia, Vancouver, BC Canada; 5 INSERM, methodS in Patient-centered outcomes and HEalth ResEarch, SPHERE, Nantes Université, Université de Tours, CHU Nantes, F-44000 Nantes, France; 6 Pharmaceutical Drugs Assessment Department, Assessment and Access to Innovation Direction, Haute Autorité de Santé, Saint-Denis, France; 7 School of Health Sciences, University of Dundee, Dundee, UK; 8 Department of Community Health Sciences, University of Calgary, Calgary, AB Canada; 9 Center for Outcomes Research and Evaluation, McGill University, Montreal, QC Canada; 10 Division of Clinical Epidemiology, Department of Medicine, McGill University Health Centre Research Institute, Montreal, QC Canada; 11 Department of Community Health Sciences, University of Manitoba, Winnipeg, MB Canada; 12 Department of Methodology and Statistics, Institute of Psychology, Leiden University, Leiden, The Netherlands; 13 Research Institute of Child Development and Education, University of Amsterdam, Amsterdam, The Netherlands

**Keywords:** Response shift, Definition, Theory, Operationalization, Method, Interpretation

## Abstract

**Purpose:**

Our aim is to advance response shift research by explicating the implications of published syntheses by the Response Shift – in Sync Working Group in an integrative way and suggesting ways for improving the quality of future response shift studies.

**Methods:**

Members of the Working Group further discussed the syntheses of the literature on definitions, theoretical underpinnings, operationalizations, and response shift methods. They outlined areas in need of further explication and refinement, and delineated additional implications for future research.

**Results:**

First, the proposed response shift definition was further specified and its implications for the interpretation of results explicated in relation to former, published definitions. Second, the proposed theoretical model was further explained in relation to previous theoretical models and its implications for formulating research objectives highlighted. Third, ways to explore alternative explanations per response shift method and their implications for response shift detection and explanation were delineated. The implications of the diversity of the response shift methods for response shift research were presented. Fourth, the implications of the need to enhance the quality and reporting of the response shift studies for future research were sketched.

**Conclusion:**

With our work, we intend to contribute to a common language regarding response shift definitions, theory, and methods. By elucidating some of the major implications of earlier work, we hope to advance response shift research.

## Introduction

The validation of patient-reported outcome measures (PROMs) over time ultimately pertains to whether the inferences we make on changes in PROM scores are justified, and subsequent actions and decisions based on those inferences are well founded. Such inferences are typically based on comparisons of responses to PROMs over time. An important source of validity evidence is information on response processes [1], which are defined as “… the mechanisms that underlie what people do, think, or feel when interacting with, and responding to (PROM items)…” [2, p. 2]. In this paper, we will use the general term ‘response processes’ to refer to these mechanisms. We will use the more specific term ‘appraisal’ (i.e. a specific set of four main types of cognitive processes proposed to describe how people respond to PROM items) only when referring to the work of Rapkin and Schwartz [3, 4]. Inferences about change over time based on the repeated administration of PROMs require that we take into account that the response processes over time may also change. There is ample evidence that respondents may not interpret and respond to PROM items in the same way at different time points as a *result of health state changes* [5, 6]. Changes in observed PROM results over time may signal changes in the measured target construct. They may also reflect a number of other response processes that influence response behaviour, including a change in the interpretation of the item, referred to as response shift [7]. Such response shifts may signal meaningful changes. However, if ignored, response shifts may threaten the validity of the inferences, actions, and decisions we will make based on the results of these PROMs over time.

The last 25 years have witnessed the burgeoning of response shift research and a concomitant increase in the heterogeneity of conceptualizations, objectives, designs, methods, and reporting of results [8]. The Response Shift – in Sync Working Group [9] was therefore established to synthesize diverse approaches wherever possible and desirable, based on a critical and comprehensive appraisal of the work to date. Among others, the Working Group focused on two interrelated topics: definitions and theoretical underpinnings [7], and operationalizations and response shift methods [10].

These two orienting works may benefit from further clarification and refinement and have implications for response shift research, either separately or combined, that have not been delineated. The aim of this paper is to advance response shift research by explicating the implications of these syntheses in an integrative way and suggesting ways for improving the quality of future response shift studies. We hereby aim to reach particularly researchers, but also health care providers and policy makers who are familiar with or want to familiarize themselves with response shift.

## Implication 1: definition

*The proposed response shift definition by Vanier and colleagues *[7]* needs further revision and clarification in relation to the definitions provided by Sprangers & Schwartz *[11]* and Rapkin & Schwartz *[3, 4]* and its implications for the interpretation of results need to be explicated.*

### Revision of the Vanier et al. [7] definition

The key definitions of response shift, provided by Sprangers and Schwartz [11], Rapkin and Schwartz [3, 4], and Vanier et al. [7] based on Oort [12, 13], are listed in Box 1. According to these definitions, measures that are particularly susceptible to response shift pertain to one’s self-evaluation [11], evaluation-based PROs [3, 4], and evaluation-based self-reports [7]. Hence, all definitions pertain to evaluations of oneself, of which PROs are a subset. We propose expanding the definition of Vanier et al. [7] to include any subjective evaluation requiring idiosyncratic criteria [3]. This would imply that response shift can also occur in proxy evaluations of other persons (e.g. patients, children) or objects (e.g. aesthetic evaluations of art). The definition can also be strengthened by including the sentence that Vanier et al. [7, p. 3316] formulated separately, in the definition itself, namely that response shift is the consequence of ‘a change in the meaning of one’s self evaluation of a target construct’. Finally, the definition can be written more precisely, highlighting that response shift is an effect, resulting in:

“Response shift is an effect on observed change that cannot be attributed to target change because of a change in the meaning of the subjective evaluation of the target construct.”

**Box 1:** Previous and new response shift definitions

• **Sprangers and Schwartz** [11] proposed the following working definition of response shift: “a change in the meaning of one's self-evaluation of a target construct as a result of: (a) a change in the respondent's internal standards of measurement (scale recalibration, in psychometric terms); (b) a change in the respondent's values (i.e. the importance of component domains constituting the target construct); or (c) a redefinition of the target construct (i.e. reconceptualization)” [11, p. 1508].	
• **Rapkin and Schwartz** [3, 4] defined response shift as the residual change score or the discrepancy between expected and observed change that can be explained by changes in appraisal, after taking into account standard influences (i.e. demographic and clinical characteristics generally considered important to quality of life (QOL) [4, p. 4].	
• **Following Oort** [12, 13], **Vanier et al.** [7] consider response shift to be a special case of violation of the principle of conditional independence (PCI) when observed change is not fully explained by target change”. They added that they assumed response shift to be the consequence of ‘a change in the meaning of one’s self evaluation of a target construct’ …” [7, p. 8].	
• **In the current paper**, we define response shift as an effect on observed change that cannot be attributed to target change because of a change in the meaning of the subjective evaluation of the target construct This definition can be operationalized as a special case of the violation of the principle of conditional independence (PCI): Response shift is present when there is a discrepancy between: (a) observed change conditioned only on change in the target construct (i.e. observed change directly reflects target change) and (b) the observed change conditioned on the target construct and other variables that explain variation in observed change (e.g. adaptation to a new health state) [7]. This definition refers to the special case where there is response shift only if the violation of the PCI is caused by a change in the meaning of the subjective evaluation of the target construct.	

Note that observed change is change in the scores on the measurement instrument (e.g. a PROM), target change is change in the targeted construct or intended outcome (e.g. a PRO), and change in meaning of subjective evaluation refers to change in response processes (e.g. recalibration [11]) when responding to the items of the measurement instrument.

This definition coincides with the formal definition of response shift, where response shift is defined as a special case of a violation of the principle of conditional independence (PCI), which can be phrased in mathematical terms [7, 13]. There are many possible causes of violations of the PCI. The current definition refers to the special case where the violation of the PCI is caused by a change in the meaning of the subjective evaluation of the target construct. Only then there is response shift (see Box 1).

### Agreements and differences among the definitions

Building on prior work by Golembiewski et al. [14] and Howard et al., [15], Sprangers and Schwartz [11] conceptualized response shift as a change in meaning of one’s self-evaluation. Conversely, Vanier et al. [7] define response shift at the *measurement* level as a discrepancy between observed and target change, as a special case of violation of the PCI, if it is *caused* by a change in the meaning of subjective evaluation. Hence, whereas change in meaning of one’s self-evaluation *is* response shift according to the conceptual definition by Sprangers and Schwartz [11], it is a *cause* of response shift according to Vanier et al. [7]. Clearly, such a discrepancy between observed and target change may not always be caused by a change in the meaning of a subjective evaluation, but for example by social desirability responding or effort justification. In those cases, it will not be considered response shift, which is consistent with the conceptual definition. Hence, as discussed by Vanier et al. [7] and Sébille et al. [10], and as indicated above, a discrepancy between observed and target change is considered a necessary condition and change in meaning of subjective evaluation a sufficient condition for the occurrence of response shift. For example, within latent variable frameworks, a lack of longitudinal measurement invariance and presence of longitudinal differential item functioning (DIF) provide evidence of PCI violation and could be considered a necessary but not sufficient condition for response shift to occur.

The response shift definitions provided by Rapkin & Schwartz [3, 4] and Vanier et al. [7] are comparable in that they both refer to a discrepancy between observed and expected change [3, 4] or target change [7] that is caused by changes in appraisal [3, 4] or changes in meaning of subjective evaluation [7]. The major difference is that the definition of Rapkin & Schwartz [3, 4], refers to an empirical study where expected change is dependent on the variables measured and change in meaning is assessed with a particular measure assessing appraisal. Conversely, Vanier et al. [7] employ a formal definition based on violation of the PCI, which is applicable to any method that can detect such discrepancies and where change in meaning can be assessed in multiple ways.

Situations might occur where a change in meaning of the subjective evaluation does not cause a discrepancy between observed and expected [3, 4] or target [7] change. Whether plausible or empirically detectable, this situation would still be considered response shift according to the conceptual definition [11], but not according to the two definitions by Rapkin & Schwartz [3, 4] and Vanier et al. [7] where these discrepancies are the *conditio sine qua non.*

We suggest a possible way to reconcile the two previous definitions [3, 4, 11] and the current definition: *how* response shift occurs (i.e. via changes in response processes, e.g. recalibration) may take place in respondents’ minds, but may be *revealed* in the violation of the PCI when there is a discrepancy between target and observed change caused by a change in the meaning of the subjective evaluation of the target construct.

## Implication 2: theory

*The proposed theoretical model by Vanier *et al*. *[7]* requires further explanation in relation to previous theoretical models *[3, 11]* and its implications for formulating research objectives need to be highlighted.*

### Recalibration, reprioritization, and reconceptualization

In the conceptual definition of response shift [11], a change in the *meaning* of one’s self-evaluation of a target construct results from recalibration, reprioritization, and/or reconceptualization. They were also referred to as the three types of response shift and were thus conceived as the causes or *why* there is change in meaning. The definitions of Rapkin and Schwartz [3, 4] and Vanier et al. [7] do not explicitly include these three types of response shift.

Rapkin and Schwartz [3] do not depict the three types of response shift in their model but rather intend their appraisal measures to operationalize them. Changes in appraisal may indicate a change in standards of comparison (recalibration), combinatory algorithm and/or sampling strategy (reprioritization), and frame of reference (i.e. reconceptualization). Vanier et al. [7] did include the three types of response shift in their theoretical model, but removed them from the ‘why’ (response shift can occur), and subsumed them under the ‘*how*’, i.e. how response shift, or the violation of the PCI due to change in meaning, can occur. Hence, the relevant question would be ‘How does response shift occur?’ and the answers could be “Through recalibrating the scale, reprioritizing the relative importance of the components constituting the target construct and/or reconceptualizing the meaning of the target construct itself”. These ways through which response shift can occur are not exhaustive as response shift can also occur via other response processes that induce or reflect change in meaning of the subjective evaluation, e.g. changes in response selection to normalize health state change [5, 6, 16].

In Vanier et al.’s [7] theoretical model, the *why* or the explanation of the violation of the PCI due to change in meaning, needs to be sought in a broader explanation of why people react to changing conditions as the underlying cause of response shift. Hence, all possible theories explaining such reactions are subsumed under the *why*, e.g. theories on cognitive homeostasis, set points, meaning making, or regaining control. This distinction is particularly important as it allows for linkages with relevant fields, such as health psychology theories and approaches that are critical to advancing response shift research [17].

### Adaptation and response shift

The response shift theoretical model proposed by Vanier et al. [7] can further provide insights as to why adaptation to changing health and response shift are distinct concepts despite the fact that they are frequently confused or used interchangeably in the literature. Here we view adaptation as the lay term to what we have defined as mechanisms, i.e. behavioural, cognitive, and affective processes to accommodate health state change [7, 11]. Hence, adaptation may refer to any variable subsumed under mechanisms, e.g. coping, social comparisons, or spiritual engagement.

The theoretical model [7, p. 3317] shows that adaptation to changing health may induce response shift only if this mechanism has an additional effect on observed change that cannot be explained by its effect on target change due to change in meaning. This happens when adaptation not only affects the responses to the PROM at follow-up through its influence on the target construct at follow-up but also directly (i.e. through path M2 [7, p. 3317]). Consequently, the general distinction is that response shift is an effect that may be caused by adaptation. It should be noted that adaptation can take place without inducing response shift. This is the case when adaptation influences the level of the target construct, and through this influence, indirectly the responses to the PROM at follow-up (i.e. through paths M1 and TC2 1) [7, p. 3317]. For example, seeking pain medication may help an individual to experience less pain over time, or taking a course in mindfulness may help a person to better cope with debilitating fatigue, without affecting the meaning of the response scales of pain and fatigue, respectively. Response shift can also occur when the catalyst directly affects the responses to the measure at follow-up (i.e. through path C3) [7, p. 3317]. For example, when the catalyst is an acute and severe shock, e.g. a car accident or emergency surgery. Hence, adaptation and response shift are distinct phenomena and they do not need to co-occur.

An implication of this distinction is to be careful and precise about the objective of an empirical study. For example, is one primarily interested in the influence of adaptation to illness on changes in the level of pain or fatigue, or in detecting response shift in the assessment of pain or fatigue over time? One should keep in mind that these two objectives are not mutually exclusive. If one would adopt our definition and model where response shift is a possible effect of adaptation (i.e. mechanisms) on observed change that cannot be attributed to target change, due to change in meaning, another implication would be to avoid referring to interventions or treatments as designed to induce a positive response shift. Rather, such interventions or treatments are meant to stimulate adaptation, which in turn may cause response shift at the measurement level, or not.

## Implication 3: methods

*The proposed list of alternative explanations per method in Sébille *et al*. *[10]* would benefit from extension and further implications for response shift detection and explanation need to be delineated. The diversity of the methods also has implications for response shift research that warrants attention.*

### Change in meaning of subjective evaluation

The main finding of the review by Sébille et al. [10] was that for all methods, response shift results cannot be accepted at face value and steps need to be taken to rule out alternative explanations or make them less likely. Vanier et al. [7] have provided a list of phenomena that are related to but distinct from response shift, which may influence responses to PROMs. Some of these can be considered alternative explanations of response shift and may be applicable to a range of methods (Table 1 [7, p 3312–5]). Sébille and colleagues [10] have listed the major alternative explanations for each method specifically. We bring our earlier work a step further by listing additional alternative explanations for each method, without claiming to be exhaustive (Table 1). One additional alternative explanation merits particular attention. According to all three definitions, the key to response shift is that a change in meaning of a subjective evaluation is at stake [3, 7, 11]. This implies that response shift methods should be able to detect a change in meaning. However, none of the extant quantitative methods are able to attribute unequivocally their results to change in the meaning of subjective evaluations [10]. With this perspective, all quantitative methods provide the necessary but not the sufficient conditions for response shift. An exception would be the appraisal method as it directly targets change in meaning of subjective evaluations. However, it is doubtful whether the derivatives of the original Quality of Life Appraisal Profile, version 2 [18] and the Brief Appraisal Inventory [19] as employed are able to assess appraisal, as these measures conflate appraisal of quality of life (QoL) with QoL itself and adaptation [10, 20]. Moreover, it is unlikely that an appraisal measure administered at the end of an entire set of various questionnaires would be able to assess the response processes of all these items [5, 6]. Further research, particularly qualitative research, on understanding (causes of) appraisal or more generally, response processes, is needed [21, 22].

**Table 1:** Exploring and making alternative explanations less plausible per response shift method

Methods	Alternative explanations based on [10]	Exploring and making alternative explanations less plausible
Design-based Methods^a^		
Then-test [15]	– Differences between pretest and then-test scores can also be due to response biases such as effort justification and social desirability.	– The more respondents invest in an intervention or the more desirable a certain outcome is, the more likely this will occur.
		– If this is the case, one might include a social desirability assessment questionnaire, preferably situation-specific. Examine whether pretest–then-test differences are larger (or smaller) in patients reporting higher levels of social desirability responding than in those reporting lower levels. – In comparative studies, employ (placebo) control condition, if possible.
	– Given the need for retrospection, this method is also prone to recall bias and implicit theories of change.^b^	– Ensure the baseline event is a salient moment for patients to increase the possibility they will remember it, e.g. by involving patients in the design of the study.
		– Compare the then-test results with those of an independent approach that cannot be affected by recall bias, e.g. statistical methods. See further [38]. – An earlier suggested method to explore recall bias included the administration of the same outcome measure at follow-up, but with the instruction to recall the baseline scores (rather than providing a re-evaluation of one’s functioning at baseline). However, this may not be a sound approach, given that response shift itself may induce recall bias. That is, given the new standard, respondents may not even be able to recall their previous responses that were given from a previous standard.
	– Without further data, it is uncertain whether the results can be attributed to a change in meaning of the subjective evaluation.	– Conduct qualitative research to understand response processes. This may include cognitive interviews with (a subsample of) respondents inviting them to reflect on their responses, starting with open, non-leading questions. For example, “If you compare the answers you have just provided (i.e. then-test) with those you gave…weeks ago (i.e. pretest), can you tell me something about it? Are the answers you have just provided generally in agreement with the answers you gave …weeks ago? Are there answers that are in disagreement? Can you amplify your answer? Can you give an example?” [39, p. 712]. Think aloud interviews with verbal probing techniques may also be conducted at different assessments to enable comparisons of respondents’ cognitive processes over time [22, 30, 31].
Appraisal method [3]	– The extant appraisal measures (e.g. the Brief Appraisal Inventory assessing health worries, concerns, goals, mood, and spirituality) do not distinguish among appraisal of QoL, QoL itself, adaptation, and response shift.	– While this questionnaire may generate new information, one should be aware that the responses will not give information on how respondents appraised a completed questionnaire but rather how they rate different aspects of their lives. However, change in people’s lives is not equivalent to change in response processes. Other measures may need to be devised or interviews may need to be held.
	– Given the need to retrospect on the way respondents completed questionnaire items, this method is prone to response bias such as recall bias and social desirability bias.	– Provided appraisal can be measured validly with a questionnaire [20], administer such a measure after completion of a limited number of related items, e.g. one questionnaire or one domain within a questionnaire. This procedure would also allow the assessment of variability of appraisal across items/domains as previously documented [6].
Semi-structured interview [21]	– Recall bias and implicit theories of change^b^ can be introduced if interview questions ask to reflect on the past.	– Ruling out alternative explanations would need to be done in a narrative way. One may probe about patients’ explanations regarding perceived change or stability. Be alert for signs of recall bias (e.g. confusion about the timing or inconsistencies in placing events in time). Recall bias can be examined when a second interview is being conducted as one may compare the recalled answers with the previously given answers when they should be similar (e.g. events).
	– Respondents may indicate change that could be interpreted as response shift but which in fact is enforced by the interview context (e.g. response biases such as demand characteristics, social desirability responding).	– Be alert for the influence of the interview context. Key is to be as open and non-judgmental as possible, and again, ask for explanations of respondents’ answers. Also be aware of inconsistencies as they can be meaningful and revealing in addition to being indicative of noise.
	– Response shift may remain undetected when respondents are not capable of reflection or verbalization.	– The interviewer may need to make a subjective judgement about whether the respondent is not capable of such reflection or verbalization. This finding may impact the conclusions, which needs to be discussed.
Vignettes [40]	– If vignettes describe health states outside respondents’ experience and knowledge, change in ratings over time may be caused by factors that are irrelevant to the vignettes.	– Make sure that the vignettes are relevant to the patient population prior to their use. The construction and validation of the vignettes may need to involve interviews or focus groups with patients of the target group.
	– Without further data, it is uncertain whether the results can be attributed to a change in meaning of the subjective evaluation.	– An interview would need to be conducted with (a subsample of) respondents. The first part of the interview needs to be aimed at ascertaining whether (lack of) changes in responses over time reflect target change or rather noise or other causes. Then respondents can be invited to reflect on their responses to the vignettes in an open, non-leading way, and to explain why they gave those responses. Previous responses to the vignettes may need to be provided to enable respondents to make such comparisons.
Individualized Methods		
Schedule for the Evaluation of Individual Quality of Life (SeiQol) [41] Patient Generated Index [42]	– Change in weights (reprioritization) may be an artefact of the calculation method as they need to add up to 100 (or to 12 or 14 imaginary points for the PGI). A decrease in the relative importance of one cue implies increases in the relative importance of other cues.	– Given this operationalization, be aware that the absolute and relative changes in weights need to be interpreted with caution.
	– Change in domain content (reconceptualization) may be caused by forgetting to nominate a domain previously mentioned (recall bias), not listing a domain that has improved, mentioning a different domain due to implicit theory of change^2^ or mentioning a similar domain at a different level of abstraction.	– All these causes will make it difficult to determine whether the domains mentioned over time are similar or dissimilar. Conducting an interview will reveal what patients had intended with their responses. An alternative is to provide the domains mentioned at baseline at follow-up and let respondents indicate whether the previously mentioned domains still hold or need to be changed. The possible, unintended influence of this approach on the results needs to be discussed.
	– If used at the individual level, changes in ranking or content of domains may be attributed to chance fluctuations, such as changes in mood or just measurement error.	– Again, only with an interview one may be able to distinguish target change from noise.
Latent Variable Models		
Structural Equation Models (SEM) [12]	– Misspecification of the measurement model (e.g. ignoring multidimensionality, response dependence over time^c^).	– Make sure the model is consistent with the data by examining model fit using several model fit indices and examining residual dependencies.
	– Inter-relations between the different forms of response shift: reprioritization may in fact reflect non-uniform recalibration and vice-versa.	– Use substantive arguments (e.g. theoretical notions, clinical insight, and/or common sense) to decide which type of response shift is most likely.
	– Change in residual variances (non-uniform recalibration) can also be due to change in intercepts (uniform recalibration) or in factor loadings (reprioritization) going in different directions.	– Change in residual variances may be caused by heterogeneity in a sample. To examine heterogeneity, covariates can be incorporated, based on theory and clinical insight, to test whether changes in intercepts or factor loadings differ in direction and/or magnitude across subgroups.
	– Induced violations of the PCI may occur due to missing data.	– Sensitivity analyses (e.g. imputation by specifying the imputation models) can be used to assess the robustness of the results to missing data. Careful examination of missing data patterns is essential (e.g. missingness over time may not be random and could be correlated with a response shift effect) [43]. Different approaches for handling missing data can be used, e.g. robust full information maximum likelihood estimates. However, to date, there is no consensus regarding the best method to handle missing data [44].
	– Without further data, it is uncertain whether the results can be attributed to a change in meaning of the subjective evaluation.	– Qualitative research about response processes, which may include interviews with (a subgroup of) respondents, are needed to understand whether the changes in responses to PROMs are attributable to change in meaning of the subjective evaluation, or something else. One way is to conduct interviews at each measurement occasion about the response processes (e.g. using think-aloud interviews, or verbal probing techniques conducted after completion of a questionnaire aimed at understanding why respondents chose the answers they gave) [22, 30, 31]. Another way is to conduct interviews only at follow-up and give back the answers respondents provided at baseline and invite them to reflect on the comparison of these responses (see also interviews for then-test and vignettes).
		Other, circumstantial evidence may also be sought.
Item Response Theory (IRT) [45]/Rasch Measurement Theory (RMT) [46]	– Misspecification of the measurement model (e.g. ignoring multidimensionality, item response dependence over time^c^).	– Make sure the model is consistent with the data at each measurement occasion by examining model fit using, e.g. Lagrange multiplier tests [47]. Test the assumption of local independence across time points [48].
	– Inter-relations between the different forms of response shift: Reprioritization may in fact reflect non-uniform recalibration and vice-versa (only IRT, not RMT).	– Use substantive evidence (e.g. theory, clinical insight, common sense) to decide which type of response shift is most likely.
	– Differential change in difficulty parameters (non-uniform recalibration) can also be due to uniform recalibration (or reprioritization for IRT) response shifts going in different directions.	– Again, this confusion may be caused by heterogeneity in a sample. Incorporate covariates, based on theory and clinical insight, to test whether changes in item parameters differ in direction and/or magnitude across subgroups.
	– Induced violations of the PCI may occur due to missing data.	– Sensitivity analyses (e.g. imputation by specifying the imputation models) can be used to assess the robustness of the results to missing data. Careful examination of missing data patterns is essential (e.g. missingness over time may not be random and could be correlated with a response shift effect) [43]. The robustness of IRT/RMT models to missing data still needs to be assessed for response shift detection.
	– Without further data, it is uncertain whether the results can be attributed to a change in meaning of the subjective evaluation.	– Conduct qualitative interviews with (a subgroup of) respondents to understand whether the changes in responses to PROMs are attributable to change in meaning of the subjective evaluation, or something else (see further under SEM).
Regression methods without classification		
Relative Importance Analysis [49]	– Relative importance of component domains is sensitive to non-normal data distributions and multi-collinearity when the analysis is conducted using discriminant analysis and logistic regression, respectively, leading to false rank ordering of the domains and false detection of reprioritization response shift.	– Start with checking the distributional assumptions for the domains and transform the data to normality, wherever appropriate when using discriminant analysis. Inspect multi-collinearity using for example the Variance Inflation Factor (VIF) when using logistic regression.
	– Change in relative importance weights or ranks may be due to the existence of more than two observed subgroups (i.e. heterogeneity due to presence of latent groups).	– As this method requires the a priori identification of two independent groups, seek the strongest evidence possible to form the two mutually exclusive subgroups, using theory, clinical knowledge, and common sense.
	– Artificial rank ordering of the domains and false detection of reprioritization response shift may be affected by missing data and mechanisms of missingness.	– Sensitivity analyses (e.g. imputation by specifying the imputation models) can be used to assess the robustness of the results to missing data. Careful examination of missing data patterns is essential (e.g. missingness over time may not be random and could be correlated with a response shift effect) [43].
	– Without further data, it is uncertain whether the results can be attributed to a change in meaning of the subjective evaluation.	– Conduct qualitative interviews with (a subgroup of) respondents to understand whether the changes in responses to PROMs are attributable to change in meaning of the subjective evaluation, or something else (see further under SEM).
Regression methods with classification		
Classification and Regression Tree (CART) [50]	– This method might be prone to model overfitting leading to false detection of response shift.	– Tree pruning or cross validation should be used to avoid overfitting.
	– If the classification variable (e.g. clinical status) is measured with measurement error or if it is unrelated to the PROM, spurious inconsistent changes in PROM scores and in the classification variable may occur, leading to false detection of recalibration response shift.	– Employ valid and reliable instruments to measure the classification variable. Use theory and clinical knowledge to identify a priori hypothesized relationship between changes in PROM scores and changes in the classification variable.
	– A selective bias towards covariates with many possible splits (i.e. with large numbers of values or categories) may lead to false detection of reprioritization response shift.	– Use theory, clinical insight, and common sense when interpreting the results of the partitioning. Conditional inference tree can be used to minimize this bias [51].
	– Without further data, it is uncertain whether the results can be attributed to a change in meaning of the subjective evaluation.	– Conduct qualitative interviews with (a subgroup of) respondents to understand whether the changes in responses to PROMs are attributable to change in meaning of the subjective evaluation, or something else (see further under SEM).
Random Forest Regression [52]	– The choice of average variable importance metric can affect the rank ordering of the component domains at each occasion and lead to false response shift detection.	– An alternative implementation of random forests based on a conditional inference framework can be applied [53] to obtain reliable variable importance measures.
	– When the autocorrelations within each domain is ignored, this might affect the estimated importance of each domain (i.e. average variable importance) and possibly the detection of response shift.	– Some recent developments on Repeated Measures Random Forests [54] may be used to handle correlated repeated measures.
	– Missing data may result in biased estimates of variable importance.	– A decision tree algorithm named Branch-Exclusive Splits Trees (BEST) that handles MCAR, MAR and MNAR missing data, can be used in case of missing data in predictors [55].
	– Without further data, it is uncertain whether the results can be attributed to a change in meaning of the subjective evaluation.	– Conduct qualitative interviews with (a subgroup of) respondents to understand whether the changes in responses to PROMs are attributable to change in meaning of the subjective evaluation, or something else (see further under SEM).
Mixed Models and Growth Mixture Models [56]	– Misspecification of the mixed model for predictions (e.g. misspecified predictors, interactions, covariance structure) might lead to inaccurate trajectories for the residuals from which response shift is deduced.	– Make sure clinical knowledge is taken on board when constructing the mixed model, using Directed Acyclic Graphs. Test for misspecification of the random effects distribution and their structure to improve the model, if appropriate.
	– Non-monotonic trajectory patterns of residuals suggesting response shift may be attributable to other phenomena, such as cognitive impairment.	– Use theory and clinical knowledge to assess the possible relationships between some clinical characteristics and the trajectory of residuals Provide sensitivity analyses with and without the patients having these clinical characteristics to assess the robustness of the results.
	– Biased trajectories of residuals suggesting response shift may occur due to missing data.	– Sensitivity analyses (e.g. available case analysis, imputation by specifying the imputation models, shared Parameter Mixture Model [57] can be used to assess the robustness of the results to missing data.
	– Without further data, it is uncertain whether the results can be attributed to a change in meaning of the subjective evaluation.	– Conduct qualitative interviews with (a subgroup of) respondents to understand whether the changes in responses to PROMs are attributable to change in meaning of the subjective evaluation, or something else (see further under SEM).

^a^Design-based methods require “study design changes (e.g. extra measures) needed to detect one or more types of response shift.” [10, p. 3332]. Whereas individualized methods can be subsumed under design-based methods, we list them separately as the respondent-generated content makes them unique

^b^Implicit theories of change: the current state of attribute or belief is assessed and a theory of stability or change is invoked

^c^Response dependence over time: the correlations among an individual’s responses cannot entirely be attributed to the latent variable, e.g. the probability of responding to a given item at time 2 may depend on the response given to that item at time 1, causing violation of the PCI (i.e. the items are not conditionally independent). This violation may be due to other causes than change in meaning of the subjective evaluation of the target construct, e.g. by effort justification influencing the assessment at time 2

### Exploring alternative explanations

For each method separately, we have further expanded our earlier work by providing ways to assess the plausibility that results are caused by change in meaning, exploring empirically the possible influence of all the other listed alternative explanations, and making these less likely by design or analysis where possible (Table 1). These actions are needed to make the conclusion that response shift may have occurred (more) plausible. However, additional validity evidence (e.g. qualitative research) and theoretical and/or clinical support for the interpretation of the results are also required to confirm that the results can indeed be attributed to response shift [2, 23–25].

One should be aware that in principle, we cannot know whether our results reflect the presence or absence of response shift. There are logically four possible situations based on whether response shift is present or absent and whether the methods have detected response shift or not. Clearly, particularly in the case of false positives and false negatives, alternative explanations or rebuttal arguments [23] would clarify these results. We are therefore obliged to explore the possible influence of alternative explanations in all empirical studies.

### Different purposes

The response shift methods are diverse and range from qualitative and individualized methods to design and statistical approaches [8, 10]. Given their variety, they may be useful for different purposes. A helpful distinction might be between methods that detect response shift, and methods that investigate (components of) response shift theory or aim to explain response shift for short.

If we want to *detect* response shift, then we need to operationalize the constructs that feature in the definition, and their interrelationships. We then would favour methods that are optimal for investigating violations of the PCI or discrepancies between observed and target change. Again, we need to check whether change in the meaning of people’s responses to PROM questions (i.e. subjective evaluation) is the cause of the discrepancies between observed and target change. In other words, finding their cause is part of response shift detection. Detection also concerns the scale or magnitude of the findings. This includes methods that are able to generate effect size estimates and methods that classify people as having undergone response shift or not. Finally, detection also refers to the ability to assess change over time while adjusting for response shift effects [10, Table 1]. This is key when the aim of the study is not only to detect response shift but also to assess change in the level of the target construct, adjusted for response shift.

If we want to *explain* response shift, we need to investigate (specific parts of) response shift theory and operationalize the constructs that feature in the theory, and their interrelationships. Based on the theoretical model proposed by Vanier and colleagues [7] we distinguish its components (i.e. target construct, catalyst, antecedents, and mechanisms), as well as “how”’ and “why” response shift occurs. This would imply that explanation encompasses how response shift occurs, why response shift occurs at study level via explanatory variables, and why response shift occurs at a more abstract, theoretical level by considering the underlying theories explaining the main principles behind response shift. In other words, finding the cause of response shift is part of response shift explanation. We therefore would favour methods that reveal *how* response shift can occur (i.e. through changes in response processes related to change in meaning of the subjective evaluation, including but not restricted to recalibration, reprioritization, and reconceptualization) and why response shift can occur by, for example, including explanatory variables (e.g. antecedents or mechanisms) in their models or conducting qualitative interviews.

In Table 2, the methods are classified according to these two, not mutually exclusive dimensions: response shift detection versus explanation. Here we include the extant methods and analytical practices used in response shift research. As can be seen, a number of methods can be used to both detect and explain response shift. Depending on whether a study aims at detecting/quantifying or explaining response shift, a method in those respective areas can be chosen. If a study targets both objectives, then a method combining both should be preferred.

**Table 2:** Classification of methods according to their ability to detect and explain response shift

	Detection					Explanation				
Methods	Observed change ≠ target change	Directly Attributable to change in meaning	Effect size estimates^a^	Classification of respondents^b^	Adjusting for response shift	Recalibration	Reprioritization	Reconcept-ualization	Other changes in response processes	Inclusion of explanatory variables
**Design-based methods**										
Then-test [15]	√	-	√	–	√	√	–	–	–	√
Appraisal, using QOLAP or BAP^c^ [3]	√	–	–^c^	–	–	–	–	–	√^c^	–
Semi-structured interview [21]	√	√	–	√	–	√	√	√	√^d^	√
Vignettes [40]	–	–	√^e^	–	–	–	√	–	–	√
**Individualized methods**										
Schedule for the Evaluation of Individual Quality of Life [41] Patient Generated Index [42]	–	–	√^f^	√^f^	–	–	√	√	–	√
**Latent variable methods**										
Structural Equation Modelling [12]	√	–	√	–	√	√	√	√^g^	–	√
Item Response Theory (IRT) [45] and Rasch Measurement Theory (RMT) [46]	√	–	√	–	√	√	√ (IRT) − (RMT)	√^g^	–	√
**Regression methods without classification**										
Relative Importance Analysis [49]	–	–	–	–	–	–	√	–	–	√^h^
**Regression methods with classification**										
Classification and Regression Tree (CART) [50]	–	–	–	√	–	√	√	–	–	√^h^
Random forest regression [52]	–	–	–	–	–	–	√	–	–	√^h^
Mixed models and growth mixture models [56]	√	–	√^i^	√	√^i^	–	√^i^	–	√^i^	√

– is not possible; √ is possible

^a^Effect size estimates: response shift effect size estimates, e.g. standardized mean differences of pre- and then-test differences

^b^Classification: classification of respondents into those who have and those who have not undergone response shift

^c^Appraisal, using the QoL Appraisal Profile (QOLAP) version 2 [18] or the Brief Appraisal Profile (BAP) [19]. When response shift is deduced from the percentage of residual variance explained by change in appraisal, effect sizes for impact on mean differences cannot be calculated. The appraisal method itself cannot be used to classify respondents, this is only possible if the data are subsequently analysed with statistical methods, such as CART. The appraisal method only provides a general response shift effect. Explanatory variables cannot explain response shift because response shift effects are indistinguishable from appraisal effects

^d^Semi-structured interviews: in principle, other (changes in) response processes may be revealed during interviews

^e^Vignettes: effect size estimates are based on mean change in vignette ratings

^f^Schedule for the Evaluation of Individual Quality of Life, Patient Generated Index: Effect sizes can be estimated on the basis of mean (standardized) observed changes in domain weights or intra-class correlation coefficient. On the basis of the individualized results, patients can be classified according to whether they provided evidence for response shift or not

^g^Structural Equation Modelling and IRT/RMT: Can operationalize reconceptualization if multidimensional generalizations of these frameworks are used

^h^Relative Importance Analysis, CART, Random Forest Regression: Explanatory variables can only be included when using as stratification variables in stratified analyses

^i^Mixed Models and Growth Mixture Models: Effect sizes can be calculated based on the 2-way interaction term of domain scores with time estimates. This method can adjust for response shift by integrating this interaction term in the mixed model. Reprioritization can be assessed by the effects of domain scores on global PRO scores that vary with time (i.e. interaction with time). Apart from reprioritization, these models can only assess a general response shift effect, i.e. centred residuals having a pattern of fluctuation over time deviating from zero

## Implication 4: future research

*The need to enhance the quality and reporting of response shift studies has implications for future research*.

### Quality and reporting of response shift research

There is a need to enhance the quality of response shift research, which may entail the following components. First, the study aims need to be explicitly defined to inform the study design. For example, a study may aim to detect response shift and/or to assess change adjusted for the possible occurrence of response shift. Prior to embarking on such a response shift study, one should think carefully ahead how likely it is that response shift would occur. Based on previous studies, detection of response shift does not automatically imply that it would affect the assessment of group level change [26, 27]. In those cases, one would need to weigh the value of possibly finding response shift against the extra effort needed to find it (i.e. designing, collecting data, analysing, and reporting). If the balance tends to be negative, then it might be better not to assess response shift. If the aim is to investigate (parts of) response shift theory or explain response shift per se, then one would also need to think carefully ahead to conscientiously design the study (e.g. timing of assessments, collecting the requisite data, using the appropriate methods) such that response shift can be demonstrated if it is present. In other words: researchers are advised to do it well or not.

Second, we would like to highlight that even when a researcher is not interested in explaining response shift per se, but rather aims to assess change adjusted for response shift, the response shift itself can point to meaningful changes. Similarly, Zumbo [28] advocated an explanation-focused approach to DIF, aimed to provide explanations of why DIF has occurred. In other words, response shift itself may reveal meaningful information in any type of study.

Third, there is need for intentional use of different response shift methods dependent on the research objective and context. Methods can either focus on detection or explanation, standardization, or exploration, and adopt a nomothetic (i.e. focused on populations or groups of people) or idiographic orientation (i.e. focused on individual differences). These approaches are all needed to advance response shift research. Moreover, applying different methods to the same data where possible rather than using a single approach would avoid overconfidence in the results and ‘model myopia’ [29].

Fourth, one particular method, however, may need to be used more frequently: qualitative methods. As indicated before, none of the extant quantitative methods can unequivocally ascribe their results to change in meaning of the self-evaluation as a cause of discrepancies between observed and expected or target change. As indicated in Table 1, qualitative interviews are recommended alongside the quantitative methods to provide insight into response processes in relation to response shift [30, 31]. Particularly cognitive interviewing and think-aloud methods may shed light on how respondents interpret and respond to PROM items and whether the underlying response processes remain equivalent over time [6, 31]. Qualitative research is also needed to develop tools for measuring changes in the meaning of subjective evaluations or changes in response processes, which can be used across studies to enhance cross-study comparability. Such measures may include an interview protocol that is applicable to a range of studies or quantitative measures that ideally could be used as an explanatory variable in a statistical model, which most quantitative methods allow [10, Table 1, pp. 3328–32] (see also Table 2 of current paper). However, construction of such a quantitative measure is far from straightforward, if possible at all, given the concerns raised to the appraisal measures [20]. Finally, qualitative methods may play a pivotal role in ongoing theoretical development.

Fifth, whereas most response shift research is practical and empirically focused, researchers are encouraged to ground their studies in a theoretical framework. For example, the theoretical model provided by Vanier et al. [7], based on explicated assumptions, related to ontology (what is response shift?) and epistemology (how do we learn about response shift and how is it different from other phenomena?), may be useful. To stimulate empirical research, Vanier and colleagues [7], electronic appendix] have provided some examples on how to empirically test (parts of) their response shift model. Other theoretical frameworks can be used, including those of Rapkin and Schwartz [3, 4] and Oort et al. [32].

Sixth, to enhance the quality of research into response shift and to safeguard against false-positive findings and publication bias, we would like to encourage researchers to use pre-registrations [33] or registered reports [34, 35]. Both formats distinguish between prediction and postdiction. Whereas the former pre-registration entails posting the protocol and analysis plan to an independent registry (timestamped at a time before the analyses can commence), a registered report is a paper accepted before the start of data collection, focusing on the study’s theoretical foundation and a prospectively planned research protocol including methods and analysis plan [34]. The subsequent paper including the results, will be accepted provided the protocol was followed (or deviations are justified) and the conclusions are sound. The results themselves (i.e. insignificant or not) will not affect the final editorial decision [34, 35].

Seventh, the quality of the reporting of studies may benefit from improvement. The work on the synthesis of the quantitative response shift research [9] was hindered by the many studies that did not provide the requisite data to enable such a synthesis. In addition, in some studies, the operationalization of response shift was ambiguous (e.g. due to conflation with measuring adaptation). A list of reporting recommendations, based on a Delphi study and endorsed by all stakeholders, including editorial boards, may be helpful. To avoid too restrictive reporting recommendations, their purpose would need to be made explicit. For example, reporting recommendations may differ for studies aimed to detect, explain, or understand response shift (e.g. qualitative studies).

Last but not least, the planning of future studies and a future research agenda may benefit from being co-led by people living with the particular condition, carers, and other stakeholders. Patients and other stakeholders provide unique insights from their perspective that may ensure that the topics of greatest importance are advanced [36]. Moreover, such engagement may enhance the quality of the study, including, for example, the study design, outcome selection, patient recruitment strategies, patient enrolment rates, and the credibility of the findings [37]. Finally, integrating diverse contributions would yield “results that go beyond the ‘average treatment effects’” as they are pertinent to specific groups of patients [36, p. 1588].

## Epilogue

We consider response shift itself to provide meaningful information that improves our understanding of change over time in PROs. The key is that our inferences, decisions, and actions made on longitudinal PROM data must consider the possibility that measurements of change over time may be influenced by response shift. However, a repeated finding is that response shift is not a self-evident or easy to understand term and many researchers attach different meanings to it. With the Response Shift – in Sync initiative, we aimed to contribute to a common language regarding definition, theory, and methods. By further elucidating and specifying this work, we hope to advance response shift research. We also intended to provide the logical or possible implications of our earlier work. Clearly, the current work is part of an ongoing process where the sketched implications are open for debate and further improvement.

Although we believe a common framework could be helpful, our goal is to promote the development and testing of theoretical frameworks and methods. We intentionally have not promoted one type of response shift method over another, as favouring one method would devalue other approaches. We believe all response shift approaches are needed to advance response shift research.

## Data Availability

Not applicable.

## References

[1] American Educational Research Association American Psychological Association, National Council on Measurement in Education (2014). Standards for educational and psychological testing.

[2] Zumbo BD, Hubley AM (2017). Understanding and investigating response processes in validation research.

[3] Rapkin BD, Schwartz CE (2004). Toward a theoretical model of quality-of-life appraisal: Implications of findings from studies of response shift. Health and Quality of Life Outcomes.

[4] Rapkin BD, Schwartz CE (2019). Advancing quality-of life research by deepening our understanding of response shift: A unifying theory of appraisal. Quality of Life Research.

[5] Bloem EF, van Zuuren FJ, Koeneman MA, Rapkin BD, Visser MRM, Koning CCE, Sprangers MAG (2008). Clarifying quality of life assessment: Do theoretical models capture the underlying cognitive processes?. Quality of Life Research.

[6] Taminiau-Bloem EF, Van Zuuren FJ, Koeneman MA, Rapkin BD, Visser MRM, Koning CCE, Sprangers MAG (2010). A ‘short walk’ is longer before radiotherapy than afterwards: A qualitative study questioning the baseline and follow-up design. Health and Quality of Life Outcomes.

[7] Vanier A, Oort FJ, McClimans L, Ow N, Gulek BG, Böhnke JR, Sprangers MAG, Sébille V, Mayo N, The Response Shift-in Sync Working Group (2021). Response shift in patient-reported outcomes measures: A formal definition and a revised model. Quality of Life Research.

[8] Sajobi TT, Brahmbatt R, Lix LM, Zumbo BD, Sawatzky R (2018). Scoping review of response shift methods: Current reporting practices and recommendations. Quality of Life Research.

[9] Sprangers MAG, Sajobi T, Vanier A, Mayo NE, Sawatzky R, Lix LM, Oort FJ, Sébille V, Response Shift—in Sync Working Group (2021). Response shift in results of patient-reported outcome measures: a commentary to The Response Shift-in Sync Working Group initiative. Quality of Life Research.

[10] Sébille V, Lix LM, Ayilara OF, Sajobi TT, Janssens CAJW, Sawatzky R, Sprangers MAG, Verdam MGE, The Response Shift—in Sync Working Group (2021). Critical examination of current response shift methods and proposal for advancing new methods. Quality of Life Research.

[11] Sprangers MAG, Schwartz CE (1999). Integrating response shift into health-related quality of life research: A theoretical model. Social Science and Medicine.

[12] Oort FJ (2005). Using structural equation modeling to detect response shifts and true change. Quality of Life Research.

[13] Oort FJ (2005). Towards a formal definition of response shift (in reply to GW Donaldson). Quality of Life Research.

[14] Golembiewski RT, Billingsley K, Yeager S (1976). Measuring change and persistence in human affairs: Types of change generated by OD designs. The Journal of Applied Behavioral Science.

[15] Howard GS, Ralph KM, Gulanick NA, Maxwell SE, Nance DW, Gerber SK (1979). Internal invalidity in pretest-posttest self-report evaluations and a re-evaluation of retrospective pretests. Applied Psychological Measurement.

[16] Tourangeau R, Rips LJ, Rasinski KA (2000). The psychology of survey response.

[17] Skolasky RL (2021). In Sync Working Group response-shift. Quality of Life Research.

[18] Rapkin BD, Garcia I, Wesley M, Zhang J, Schwartz CE (2017). Distinguishing appraisal and personality influences on quality of life in chronic illness: Introducing the quality-of-life appraisal profile version 2. Quality of Life Research.

[19] Rapkin BD, Garcia I, Wesley M, Zhang J, Schwartz CE (2018). Development of a practical outcome measure to account for individual differences in quality-of-life appraisal: The brief appraisal inventory. Quality of Life Research.

[20] Verdam MGE, Oort FJ (2019). Conceptual and methodological considerations regarding appraisal and response shift. Quality of Life Research.

[21] Beeken RJ, Eiser C, Dalley C (2011). Health-related quality of life in haematopoietic stem cell transplant survivors: A qualitative study on the role of psychosocial variables and response shifts. Quality of Life Research.

[22] Taminau-Bloem EF, Schwartz CE, van Zuuren FJ, Koeneman MA, Visser MRM, Tishelman C, Koning CCE, Sprangers MAG (2016). Using a retrospective pretest instead of a conventional pretest is replacing biases: A qualitative study of cognitive processes underlying responses to thentest items. Quality of Life Research.

[23] Hawkins M, Elsworth GR, Nolte S, Osborne RH (2021). Validity arguments for patient-reported outcomes: Justifying the intended interpretation and use of data. Journal of Patient-Reported Outcomes.

[24] Hawkins M, Elsworth GR, Osborne RH (2018). Application of validity theory and methodology to patient-reported outcome measures (PROMs): Building an argument for validity. Quality of Life Research.

[25] Weinfurt KV (2021). Constructing arguments for the interpretation and use of patient-reported outcome measures in research: An application of modern validity theory. Quality of Life Research.

[26] Oreel TH, Nieuwkerk PT, Hartog ID, Netjes JE, Vonk ABA, Lemkes J, van Laarhoven HWM, Scherer-Rath M, Henriques JPS, Oort FJ, Sprangers MAG, Verdam MGE (2021). Response shift after coronary revascularization. Quality of Life Research.

[27] Verdam MGE, Van Ballegooijen W, Holtmaat CJM, Knoop H, Lancee J, Oort FJ, Riper H, Van Straten A, Verdonck-de Leeuw IM, De Wit M, Van der Zweerde T, Sprangers MAG (2021). Re-evaluating randomized clinical trials of psychological interventions: Impact of response shift on the interpretation of trial results. PLoS ONE.

[28] Zumbo BD (2007). Three generations of DIF analyses: Considering where it has been, where it is now, and where it is going. Language Assessment Quarterly.

[29] Wagenmakers E-J, Sarafoglou A, Axel B (2022). One statistical analysis must not rule them all. Nature.

[30] Leighton JP, Tang W, Gue Q, Zumbo BD, Hubley AM (2017). Response processes and validity evidence: Controlling for emotions in think aloud interviews. Understanding and investigating response processes in validation research.

[31] Padilla J-L, Leighton JP, Zumbo BD, Hubley AM (2017). Cognitive interviewing and think aloud methods. Understanding and investigating response processes in validation research.

[32] Oort FJ, Visser MRM, Sprangers MAG (2009). Formal definitions of measurement bias and explanation bias clarify measurement and conceptual perspectives on response shift. Journal of Clinical Epidemiology.

[33] Nosek BA, Ebersole CR, DeHaven AC, Mellor DT (2018). The preregistration revolution. PNAS.

[34] Boehnke JR, Rutherford C (2020). Registered reports at “quality of life research”. Quality of Life Research.

[35] Chambers C (2019). The registered reports revolution Lessons in cultural reform. Significance.

[36] Mullins CD, Abdulhalim AM, Lavallee DC (2012). Continuous patient engagement in comparative effectiveness research. JAMA.

[37] Forsythe L, Heckert A, Margoli MK, Schrand S, Frank L (2018). Methods and impact of engagement in research, from theory to practice and back again: Early findings from the patient-centered outcomes research institute. Quality of Life Research.

[38] Schwartz CE, Sprangers MAG (2010). Guidelines for improving the stringency of response shift research using the thentest. Quality of Life Research.

[39] Sprangers MAG, van Dam FSAM, Broersen J, Lodder Wever LL, Visser M, Oosterveld P, Smets E (1999). Revealing response shift in longitudinal research on fatigue: The use of the thentest approach. Acta Oncologica.

[40] Korfage IJ, de Koning HJ, Essink-Bot M-L (2007). Response shift due to diagnosis and primary treatment of localized prostate cancer: A then-test and a vignette study. Quality of Life Research.

[41] Ring L, Höfer S, Heuston F, Harris D, O’Boyle CA (2005). Response shift masks the treatment impact on patient reported outcomes (PROs): The example of individual quality of life in edentulous patients. Health and Quality of Life Outcomes.

[42] Aburub AS, Gagnon B, Ahmed S, Rodriguez AM, Mayo NE (2018). Impact of reconceptualization response shift on rating of quality of life over time among people with advanced cancer. Supportive Care in Cancer.

[43] Sajobi TT, Lix LM, Singh G, Lowerison M, Engbers J, Mayo NE (2015). Identifying reprioritization response shift in a stroke caregiver population: A comparison of missing data methods. Quality of Life Research.

[44] Chen P, Wu W, Brandt H, Jia F (2020). Addressing missing data in specification search in measurement invariance testing with Likert-type scale variables: A comparison of two approaches. Behavior Research Methods.

[45] Guilleux A, Blanchin M, Vanier A, Guillemin F, Falissard B, Schwartz CE, Sébille V (2015). RespOnse Shift ALgorithm in Item response theory (ROSALI) for response shift detection with missing data in longitudinal patient-reported outcome studies. Quality of Life Research: An International Journal of Quality of Life Aspects of Treatment, Care and Rehabilitation.

[46] Blanchin M, Guilleux A, Hardouin J-B, Sébille V (2020). Comparison of structural equation modelling, item response theory and Rasch measurement theory-based methods for response shift detection at item level: A simulation study. Statistical Methods in Medical Research.

[47] Te Marvelde JM, Glas CAW, Van Landeghem G, Van Damme J (2006). Application of multidimensional item response theory models to longitudinal data. Educational and Psychological Measurement.

[48] Olsbjerg M, Christensen KB (2015). %lrasch_mml: A SAS macro for marginal maximum likelihood estimation in longitudinal polytomous Rasch models. Journal of Statistical Software, Code Snippets.

[49] Lix LM, Sajobi TT, Sawatzky R, Liu J, Mayo NE, Huang Y, Bernstein CN (2013). Relative importance measures for reprioritization response shift. Quality of Life Research: An International Journal of Quality of Life Aspects of Treatment, Care and Rehabilitation.

[50] Li Y, Rapkin B (2009). Classification and regression tree uncovered hierarchy of psychosocial determinants underlying quality of life response shift in HIV/AIDS. Journal of Clinical Epidemiology.

[51] Fu W, Simonoff JS (2015). Unbiased regression trees for longitudinal and clustered data. Computational Statistics and Computational Analysis.

[52] Boucekine M, Boyer L, Baumstarck K, Millier A, Ghattas B, Auquier P, Toumi M (2015). Exploring the response shift effect on the quality of life of patients with schizophrenia: An application of the random forest method. Medical Decision Making: An International Journal of the Society for Medical Decision Making.

[53] Strobl C, Boulesteix A-L, Zeileis A, Hothorn T (2007). Bias in random forest variable importance measures: Illustrations, sources and a solution. BMC Bioinformatics.

[54] Calhoun P, Levine RA, Fan J (2021). Repeated measures random forests (RMRF): Identifying factors associated with nocturnal hypoglycemia. Biometrics.

[55] Beaulac C, Rosenthal JS (2020). BEST: A decision tree algorithm that handles missing values. Computational Statistics.

[56] Mayo NE, Scott SC, Dendukuri N, Ahmed S, Wood-Dauphinee S (2008). Identifying response shift statistically at the individual level. Quality of Life Research: An International Journal of Quality of Life Aspects of Treatment, Care and Rehabilitation.

[57] Gottfredson NC, Bauer DJ, Baldwin SA (2014). Modeling change in the presence of nonrandomly missing data: Evaluating a shared parameter mixture model. Structural Equation Modeling: A Multidisciplinary Journal.

